# Do SOD2 and *SOD3* gene polymorphisms impact the oral health-related quality of life in Para athletes?

**DOI:** 10.1590/1807-3107bor-2024.vol38.0074

**Published:** 2024-08-05

**Authors:** Anna Carolina Jesus SILVEIRA, Ludmila Silva GUIMARÃES, Rodrigo VON HELD, Erlange Borges da SILVA, Flavia Maia SILVEIRA, Zair Candido OLIVEIRA NETO, Rafaela SCARIOT, Ciro WINCKLER, Erika Calvano KÜCHLER, João Armando BRANCHER, Lívia Azeredo Alves ANTUNES, Leonardo Santos ANTUNES

**Affiliations:** (a) Universidade Federal Fluminense – UFF, School of Dentistry, Postgraduate Program in Dentistry, Nova Friburgo, RJ, Brazil.; (b) Universidade Federal Fluminense – UFF, School of Dentistry, Postgraduate Program in Dentistry, Niterói, RJ, Brazil.; (c) Universidade Positivo, School of Health Science, Postgraduate Program in Dentistry, Curitiba, PR, Brazil.; (d) Universidade Federal do Paraná – UFPR, School of Health Science, Department of Stomatology, Curitiba, PR, Brazil.; (e) Universidade Federal de São Paulo – UFSP, Human Movement Science Department, Santos, SP, Brazil.; (f) Universidade Tuiuti do Paraná, Curitiba, PR, Brazil.

**Keywords:** Polymorphism, Genetic, Quality of Life, Para-Athletes, Dental Caries

## Abstract

The aim of this study was to evaluate whether polymorphisms in SOD2 and *SOD3* genes modulate the oral health-related quality of life (OHRQoL) of Para athletes with dental caries experience. The cross-sectional study included 264 Para athletes (143 in athletics, 61 in weightlifting and 60 in swimming). A trained and calibrated team recorded the decayed, missing and filled teeth index (DMFT). The Brazilian version of the Oral Health Impact Profile (OHIP-14) was used to measure OHRQoL. Genomic DNA was extracted from the athletes’ saliva, and genetic polymorphisms in the SOD2 (rs5746136 and rs10370) and *SOD3* (rs2855262 and rs13306703) genes were analyzed by real-time polymerase chain reaction. Univariate and multivariate analyses were performed. A multivariate General Linear Model analysis, adjusted for sex, revealed that the *SOD3* gene polymorphism (rs2855262) had a significant effect on the psychological disability domain [codominant (p = 0.045) and recessive (p=0.038) models]. The *SOD2* gene polymorphism (rs5746136) had a significant effect on the total OHIP-14 score [dominant model (p = 0.038)] and the psychological discomfort [dominant model (p = 0.034)] and physical disability [codominant model (p=0.037)] domains. Presence of the *SOD2* rs10370 polymorphism led to statistical differences in the total score [codominant (p = 0.026) and dominant (p = 0.023) models] and the handicap domain scores [codominant (p = 0.027) and dominant (p = 0.032) models]. Polymorphisms of the *SOD2* and *SOD3* genes may be important biomarkers of OHRQoL in Para athletes with dental caries experience.

## Introduction

Practicing sports improves health and quality of life and helps ensure the rights and social inclusion of people with disabilities.^1^ However, athletes commonly present with oral health-related problems, which can directly affect their performance and oral health-related quality of life (OHRQoL).^2^

The Paralympics is considered the third-largest sporting event in the world, following only the Olympics and the World Cup. Paralympic sports have gained such magnitude that they have sparked the interest of researchers in sports science, specifically those seeking to understand the importance of high-performance sports in the lives of Para athletes.^3,4^ In the most recent last Paralympic Games, almost 50% of all awarded medals were won by Brazilian swimming and athletic teams. However, despite these impressive results, studies on Paralympic athletes are still scarce. This is especially true for Para athletes who practice swimming and athletics.^3^

The OHRQoL has been used to measure the impact of oral diseases and disorders on individuals and society. Unlike normative clinical indicators, OHRQoL measures aim to capture the consequences of poor oral health.^5^ Furthermore, data based on patients’ self-perception of oral health and OHRQoL help complement clinical data with the patient’s perspective, which can, in turn, lead to better clinical decision-making.^6^

The Consortium for Genetics and Quality of Life Research (GeneQoL) developed a list of candidate genes that may have an impact on health-related quality of life (HRQoL).^7^ In dentistry, recent studies have identified possible genetic biomarkers for OHRQoL, such as ANKK1 (rs1800497)^8^, IL6 (rs1800795, rs1800796)^9^, IL1A (rs17561)^10^, TNF-α (rs1799964)^11^, and MTRR (rs1801394).^12^

Superoxide dismutases (SODs) are a ubiquitous family of enzymes that efficiently catalyze the dismutation of superoxide anions.^13^ *SOD2* (also known as Mn-SOD; EC 1.15.1.1) is located on chromosome 6, exists as a tetramer, and is initially synthesized containing a leader peptide which directs the manganese-containing enzyme exclusively to the mitochondria. *SOD3* (EC-SOD; EC 1.15.1.1), located on chromosome 4, exists as a tetramer containing copper and zinc and is synthesized with a signal peptide that directs it exclusively to extracellular spaces. Psychological disorders such as depression and generalized anxiety disorder have previously been associated with changes in SOD activity.^14,15^ In addition, a previous study by Lopez-Jornet and coworkers^16^ suggested a possible association between salivary biomarkers of oxidative stress and quality of life in patients with oral lichen planus.

In our previous study^10^, we observed that dental caries affected OHRQoL in Para athletes and suggested that polymorphisms in the gene encoding IL1A were potential biomarkers for OHRQoL in this population. Thus, this study aimed to evaluate whether polymorphisms of *SOD2* and *SOD3* genes are associated with OHRQoL in Para athletes with dental caries experience. The positive hypothesis is that polymorphisms of *SOD2* and *SOD3* genes modulate the impact of OHRQoL in Para athletes with dental caries experience.

## Methods

### Study type, ethical aspects, and sampling

This cross-sectional genetic study was based on guidelines from the Strengthening the Reporting of Genetic Association initiative.^17^ It was approved by the local Research Ethics Committee (number 3,261,377). All participants had access to the free and informed consent form, as previously described by Von Held et al.^10^

Adult Para athletes from swimming, weightlifting, and athletics who attended a regional competition in the city of Curitiba, Brazil were included in this study. This event was held by the Brazilian Paralympic Committee between April 12 and 14, 2019.

Athletes who did not signor who did not correctly fill out the consent form, and those with intellectual disabilities that could affect their ability to adequately answer a questionnaire, were also excluded.

A sample size calculation was performed with a power of 80% and alpha of 0.05. We assumed a mean difference of 3.5 among genotypes for the total Oral Health Impact Profile scale (OHIP-14), resulting in an estimated minimum sample of 32 patients per genotype for the comparisons.

### Data collection

#### Non-clinical data

Initially, participants answered questions about their age, gender, degree of schooling, and the sport that they practiced.

The Brazilian short form of the OHIP-14 questionnaire^18^ was chosen to assess the impact of oral health problems such as dental caries on OHRQoL through interviews conducted by a trained dentist. The OHIP-14 questionnaire consists of two questions for each of seven domains: functional limitation, physical pain, psychological discomfort, physical disability, psychological disability, social disability, and handicap, totaling 14 questions. Each question was scored on a Likert scale: 0= never; 1= rarely; 2= sometimes; 3= often; 4= always. The total score is the sum of the 14 responses and ranges from 0 to 56 points, where higher values indicate worse OHRQoL. Each of the seven domains was analyzed separately regarding its OHIP-14 values.^18^

## Clinical data

The oral examination of the Para athletes was performed by a trained team of dentists and note-takers at the competition site. The athlete was seated in a chair and examined with natural ambient light using tongue depressors and gauze.

Calibration for the clinical examination and the step-by-step theoretical and practical stages of the training was conducted by an examiner experienced in epidemiology, considered the gold standard. After calibration, the inter- and intra-examiner kappa values were greater than 0.90, indicating a high degree of data reproducibility.

The decayed, missing, and filled teeth index (DMFT), recommended by the World Health Organization (WHO)^19^, was used to evaluate the athletes’ oral health according to the presence or absence of dental caries. The numbers of decayed (D), missing (M) and filled (F) teeth (T) were evaluated, and the DMFT was calculated based on these clinical observations. Phenotypes were determined in caries-free athletes (DMFT = 0; used as the control group); athletes with dental caries experience (DMFT ≥ 1) were considered the case group.

## Deoxyribonucleic acid collection and genotyping

The genotyping analysis was performed using deoxyribonucleic acid (DNA) collected from oral cells obtained through a 60 second mouth rinse with 15 mL of saline solution. This material was stored in a 50 mL propylene tube and kept at -20ºC until DNA extraction.^20^ A spectrophotometer (Nanodrop 1000, Thermo Scientific; Wilmington, DE, USA) was used to establish the amount and purity of the DNA.

Candidate genes were chosen based on the GeneQoL recommendations, and the genome browser of the University of California at Santa Cruz (https://genome.ucsc.edu) was used to identify the polymorphisms. Polymorphisms in the *SOD2* (rs5746136 and rs10370) and *SOD3* (rs2855262 and rs13306703) genes were genotyped by real-time polymerase chain reaction using the TaqMan method. Data interpretation was performed using software provided by Applied Biosystems (Foster City, USA) for allelic discrimination. The laboratory examiners were blinded to the sample groups.

## Statistical analysis

Pearson’s chi-square test without correction was applied to evaluate the Hardy-Weinberg equilibrium and the distribution of patients according to sex, sport modality, and genotype using SPSS software (IBM SPSS Statistics for Windows, Version 25.0. Armonk, NY: IBM Corp.). Three models (codominant, dominant, and recessive) were tested for each genotype.

Multifactor Dimensionality Reduction (MDR; sourceforge.net/projects/mdr/files) was used to assess the risk of polymorphism-polymorphism interactions on the development of dental caries. The polymorphism-polymorphism interaction models were assessed using 10-fold cross-validation consistency (CVC), testing balancing accuracy (TBA), and a 1000 permutation test to determine statistical significance. The best models were identified based on a cross-validation consistency of CVC=9/10 or 10/10, TBA > 0.55, and p ≤ 0.05. Entropy values were calculated, and the MDR generated dendrograms and interaction graphs using these values.

Quality of life values were compared by OHIP in patients with DFMT>1 using the Mann-Whitney U test. In subgroup analyses, the variable for sex varied with genotype distribution. Thus, a General Linear Model (GLM) was used to evaluate the impact of genotypes on quality of life adjusted for sex (NCSS, LLC. Kaysville, Utah, USA). Statistical significance was set at p < 0.05.

## Results

Of the 616 Para athletes attending the competition, 264 (143 in athletics, 61 in weightlifting, and 60 in swimming) participated in the study. Two hundred twenty-one participants (128 in athletics, 50 in weightlifting, and 43 in swimming) had dental caries and were allocated to the case group. This group consisted of 147 men and 74 women with a mean age of 31.2 years (standard deviation (SD),11.7). Figure shows a flowchart of the genotyping analysis.

**Figure 1:**
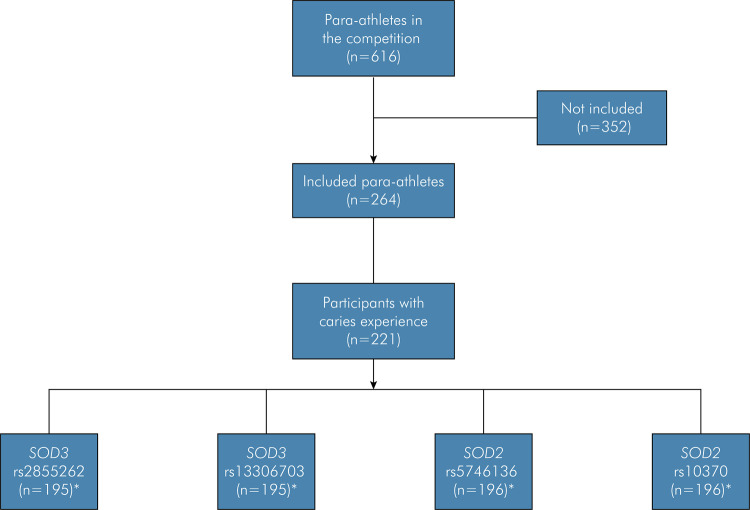
Flowchart of the genotyping analysis.

Notes: * Number of samples that amplified in the real-time PCR analysis.

The overall mean OHIP-14 score was 10.03 (SD, 8.11), whereas the median score was 8 (4–15) in the participants with dental caries experience.

The variables age, gender and sport modality were evaluated regarding the total scale and each domainof the OHIP-14 questionnaire in Para athletes with dental caries experience. A statistically significant difference was found regarding gender and the total scale of OHIP-14 (p = 0.008), physical pain (p = 0.015) and physical disability (p=0.041). Women had lower OHRQoL values than men (Table 1).

**Table 1:** The median of total scale and each domain of OHIP-14 between variables age, gender and sport modality.

Variable	GENERAL OHIP		OHIP1		OHIP2		OHIP3		OHIP4		OHIP5		OHIP6		OHIP7	

	Median (Q1–Q3)	p–value	Median (Q1–Q3)	p–value	Median (Q1–Q3)	p–value	Median (Q1–Q3)	p–value	Median (Q1–Q3)	p–value	Median (Q1–Q3)	p–value	Median (Q1–Q3)	p–value	Median (Q1–Q3)	p–value
Age (years)*																
≤ 20	7.0 (3–14)		2.0 (0–3)		2.0 (0–3)		0.0 (0–4)		0.0 (0–2)		1.0 (0–2)		0.0 (0–2)		0.0 (0–0)	0.276
≥ 21 and ≤ 40	8.0 (4–15)	0.240	0.0 (0–2)	0.057	2.0 (0–3)	0.825	2.0 (0–4)	0.077	0.0 (0–2)	0.416	1.0 (0–3)	0.140	0.0 (0–2)	0.626	0.0 (0–1)	
≥ 41	12.5 (5–16)		0.0 (0–1)		2.0 (0–3)		3.0 (0.5–5)		0.0 (0–2.5)		2.0 (0–4)		0.0 (0–3)		0.0 (0–2)	
Gender**																
Men	7.0 (2–14)	**0.008**	0.0 (0–2)	0.091	1.0 (0–2)	**0.015**	2.0 (0–4)	0.348	0.0 (0–2)	**0.041**	1.0 (0–2)	0.058	0.0 (0–2)	0.054	0.0 (0–1)	0.923
Women	12.5 (6–17)		1.0 (0–2)		2.0 (0–3)		2.0 (0–4)		0.0 (0–2)		2.0 (0–3)		1.0 (0–3)		0.0 (0–1)	
Sport modality*																
Athletics	8.0 (2–16)		1.0 (0–3)		1.0 (0–3)		2.0 (0–4)		0.0 (0–2)		1.0 (0–3)		0.0 (0–2)		0.0 (0–0)	
Weightlifting	8.0 (3–13)	0.785	0.0 (0–2)	0.101	2.0 (1–3)	0.147	2.5 (0–4)	0.874	0.0 (0–2)	0.877	1.0 (0–2)	0.375	0.0 (0–3)	0.472	0.0 (0–0)	0.409
Swimming	8.0 (4–16)		1.0 (0–2)		2.0 (0–3)		2.0 (0–4)		0.0 (0–2)		2.0 (0–3.5)		0.0 (0–2)		0.0 (0–1)	

Q1 = percentile 25, Q3 = percentile 75; *Kruskal-Wallis test, with a significance level of 0.05; **Mann-Whitney U test, Bold indicates statistical significance. OHIP1 = functional limitation, OHIP2 = physical pain, OHIP3 =psychological discomfort, OHIP4 = physical disability, OHIP5 = psychological disability, OHIP6 = social disability, OHIP7 = handicap.

Details of the genetic markers evaluated in this study are described in Table 2. The genotypic distribution of each genotype was consistent with the Hardy-Weinberg equilibrium proportions (p > 0.05).

**Table 2:** Details on the genetic biomarkers evaluated in this study.

Chromosome	Gene	SNP	Also known as	Nucleotide exchange	Global MAF	Functional consequence
4	*SOD3*	rs2855262	g.9892T>C	C > T	0.39	Non-coding transcriptional variant
		rs13306703	-	C > T	0.15	“Upstream” variant
6	*SOD2*	rs5746136	c.*441G>A	C > T	0.26	Intronic variant
		rs10370	-	T > G	0.22	Intronic variant

Notes: Information obtained from http://www.thermofisher.com, http://www.ncbi.nlm.nih.gov, and http://genome.ucsc.edu data bases. SNP: Single Nucleotide Polymorphism. MAF: Minor Allele Frequency. C: Cytosine; T: Thymine; G: Guanine.

The distributions between genotypes in the OHIP-14 total and domain scores in the codominant, dominant, and recessive models are summarized in Table 3. The rs2855262 polymorphism in the *SOD3* gene revealed a significant difference in the following domains: physical disability [recessive model (p = 0.028)], social disability [codominant (p = 0.014) and dominant (p = 0.043) models], and handicap [codominant (p = 0.034) and dominant (p = 0.043) models]. In contrast, the analysis of rs13306703 revealed significant differences in total score [codominant (p = 0.031) and dominant (p = 0.041) models] and handicap [codominant (p = 0.013) and dominant (p = 0.013) models]. In the *SOD2* gene analysis, the rs5746136 polymorphism revealed significant differences in total score [dominant model (p = 0.039)], functional limitation [codominant (p = 0.018) and dominant (p = 0.033) models], psychological discomfort [codominant (p = 0.042) and dominant (p = 0.025) models] and social disability [codominant model (p = 0.028)], while the analysis of rs10370 revealed significant differences in total score [codominant (p = 0.009) and dominant (p = 0.011) models], functional limitation [codominant (p = 0.005) and dominant (p = 0.017) models], psychological discomfort [codominant (p = 0.014) and dominant (p = 0.009) models], and social disability [codominant model (p = 0.047)].

**Table 3:** Comparison of domain values using OHIP-14 tool between genotypes.

Gene	SNP	Model	Genotypes	n	%	General OHIP				OHIP1				OHIP2				OHIP3			

						Median	Q25	Q75	p-value	Median	Q25	Q75	p-value	Median	Q25	Q75	p-value	Median	Q25	Q75	p-value
*SOD3*	rs2855262	Co-Dominant	CC	58	29.7	11.5	5.0	17.0	Ref.	1.0	0.0	2.0	Ref.	2.0	0.0	3.0	Ref.	2.0	0.0	4.0	Ref.
			CT	99	50.8	7.0	3.0	13.0	0.052	0.0	0.0	2.0	0.433	2.0	0.0	3.0	0.29	2.0	0.0	4.0	0.578
			TT	38	19.5	11.0	4.0	16.0	0.895	0.5	0.0	2.0	0.531	2.0	0.0	3.0	0.975	2.0	0.0	4.0	0.706
		Dominant	CC	58	29.7	11.5	5.0	17.0	Ref.	1.0	0.0	2.0	Ref.	2.0	0.0	3.0	Ref.	2.0	0.0	4.0	Ref.
			CT + TT	137	70.3	8.0	4.0	14.0	0.126	0.0	0.0	2.0	0.404	2.0	0.0	3.0	0.414	2.0	0.0	4.0	0.571
		Recessive	CC + CT	157	80.5	8.0	4.0	15.0	Ref.	0.0	0.0	2.0	Ref.	2.0	0.0	3.0	Ref.	2.0	0.0	4.0	Ref.
			TT	38	19.5	11.0	4.0	16.0	0.364	0.5	0.0	2.0	0.798	2.0	0.0	3.0	0.55	2.0	0.0	4.0	0.922
	rs13306703	Co-Dominant	CC	144	73.8	9.0	4.0	16.0	Ref.	0.0	0.0	2.0	Ref.	2.0	0.0	3.0	Ref.	2.0	0.0	4.0	Ref.
			CT	48	24.6	6.0	3.5	11.0	0.031*	0.0	0.0	2.0	0.381	1.5	0.0	2.0	0.221	2.0	0.0	4.0	0.419
			TT	3	1.5	12.0	1.0	21.0	0.89	2.0	0.0	2.0	0.659	2.0	1.0	3.0	0.688	3.0	0.0	4.0	0.921
		Dominant	CC	144	73.8	9.0	4.0	16.0	Ref.	0.0	0.0	2.0	Ref.	2.0	0.0	3.0	Ref.	2.0	0.0	4.0	Ref.
			CT + TT	51	26.2	6.0	3.0	12.0	0.041*	0.0	0.0	2.0	0.454	2.0	0.0	2.0	0.275	2.0	0.0	4.0	0.425
		Recessive	CC + CT	192	98.5	8.0	4.0	15.0	Ref.	0.0	0.0	2.0	Ref.	2.0	0.0	3.0	Ref.	2.0	0.0	4.0	Ref.
			TT	3	1.5	12.0	1.0	21.0	0.784	2.0	0.0	2.0	0.593	2.0	1.0	3.0	0.595	3.0	0.0	4.0	0.966
*SOD2*	rs5746136	Co-Dominant	CC	96	49	9.0	5.5	16.0	Ref.	1.0	0.0	2.0	Ref.	2.0	0.0	3.0	Ref.	3.0	0.0	5.0	Ref.
			CT	82	41.8	8.0	2.0	14.0	0.072	0.0	0.0	2.0	0.018*	2.0	0.0	3.0	0.819	2.0	0.0	4.0	0.042*
			TT	18	9.2	6.5	1.0	14.0	0.119	1.0	0.0	2.0	0.758	0.5	0.0	3.0	0.352	2.0	0.0	4.0	0.145
		Dominant	CC	96	49	9.0	5.5	16.0	Ref.	1.0	0.0	2.0	Ref.	2.0	0.0	3.0	Ref.	3.0	0.0	5.0	Ref.
			CT + TT	100	51	7.5	2.0	14.0	0.039*	0.0	0.0	2.0	0.033*	2.0	0.0	3.0	0.918	2.0	0.0	4.0	0.025*
		Recessive	CC + CT	178	90.8	8.0	4.0	16.0	Ref.	0.0	0.0	2.0	Ref.	2.0	0.0	3.0	Ref.	2.0	0.0	4.0	Ref.
			TT	18	9.2	6.5	1.0	14.0	0.257	1.0	0.0	2.0	0.719	0.5	0.0	3.0	0.299	2.0	0.0	4.0	0.377
	rs10370	Co-Dominant	TT	113	57.7	9.0	6.0	16.0	Ref.	1.0	0.0	2.0	Ref.	2.0	0.0	3.0	Ref.	3.0	0.0	4.0	Ref.
			TG	73	37.2	6.0	2.0	14.0	0.009*	0.0	0.0	1.0	0.005*	2.0	0.0	3.0	0.77	2.0	0.0	4.0	0.062
			GG	10	5.1	8.0	6.0	14.0	0.549	1.0	0.0	2.0	0.675	1.0	0.0	2.0	0.425	3.0	0.0	4.0	0.917
		Dominant	TT	113	57.7	9.0	6.0	16.0	Ref.	1.0	0.0	2.0	Ref.	2.0	0.0	3.0	Ref.	3.0	0.0	4.0	Ref.
			TG + GG	83	42.3	7.0	2.0	14.0	0.011*	0.0	0.0	2.0	0.017*	2.0	0.0	3.0	0.627	2.0	0.0	4.0	0.083
		Recessive	TT + TG	186	94.9	8.0	4.0	16.0	Ref.	0.0	0.0	2.0	Ref.	2.0	0.0	3.0	Ref.	2.0	0.0	4.0	Ref.
			GG	10	5.1	8.0	6.0	14.0	0.926	1.0	0.0	2.0	0.318	1.0	0.0	2.0	0.448	3.0	0.0	4.0	0.791
*SOD3*	rs2855262	Co-Dominant	CC	58	29.7	0.0	0.0	2.0	Ref.	2.0	0.0	4.0	Ref.	2.0	0.0	2.0	Ref.	0.0	0.0	2.0	Ref.
			CT	99	50.8	0.0	0.0	1.0	0.396	1.0	0.0	3.0	0.137	0.0	0.0	2.0	0.014*	0.0	0.0	0.0	0.033*
			TT	38	19.5	1.5	0.0	2.0	0.144	2.0	0.0	3.5	0.984	0.0	0.0	3.0	0.676	0.0	0.0	1.0	0.313
		Dominant	CC	58	29.7	0.0	0.0	2.0	Ref.	2.0	0.0	4.0	Ref.	2.0	0.0	2.0	Ref.	0.0	0.0	2.0	Ref.
			CT + TT	137	70.3	0.0	0.0	2.0	0.943	1.0	0.0	3.0	0.254	0.0	0.0	2.0	0.043*	0.0	0.0	0.0	0.043*
		Recessive	CC + CT	157	80.5	0.0	0.0	2.0	Ref.	1.0	0.0	3.0	Ref.	0.0	0.0	2.0	Ref.	0.0	0.0	1.0	Ref.
			TT	38	19.5	1.5	0.0	2.0	0.028*	2.0	0.0	3.5	0.456	0.0	0.0	3.0	0.459	0.0	0.0	1.0	0.985
	rs13306703	Co-Dominant	CC	144	73.8	0.0	0.0	2.0	Ref.	2.0	0.0	3.0	Ref.	0.0	0.0	2.0	Ref.	0.0	0.0	1.5	Ref.
			CT	48	24.6	0.0	0.0	1.0	0.085	0.0	0.0	2.0	0.091	0.0	0.0	2.0	0.145	0.0	0.0	0.0	0.013*
			TT	3	1.5	2.0	0.0	2.0	0.587	2.0	0.0	6.0	0.686	0.0	0.0	4.0	0.83	0.0	0.0	1.0	0.734
		Dominant	CC	144	73.8	0.0	0.0	2.0	Ref.	2.0	0.0	3.0	Ref.	0.0	0.0	2.0	Ref.	0.0	0.0	1.5	Ref.
			CT + TT	51	26.2	0.0	0.0	2.0	0.125	0.0	0.0	2.0	0.123	0.0	0.0	2.0	0.147	0.0	0.0	0.0	0.013*
		Recessive	CC + CT	192	98.5	0.0	0.0	2.0	Ref.	1.0	0.0	3.0	Ref.	0.0	0.0	2.0	Ref.	0.0	0.0	1.0	Ref.
			TT	3	1.5	2.0	0.0	2.0	0.481	2.0	0.0	6.0	0.616	0.0	0.0	4.0	0.887	0.0	0.0	1.0	0.893
*SOD2*	rs5746136	Co-Dominant	CC	96	49	0.0	0.0	2.0	Ref.	2.0	0.0	3.0	Ref.	1.0	0.0	3.0	Ref.	0.0	0.0	1.0	Ref.
			CT	82	41.8	0.0	0.0	2.0	0.878	1.0	0.0	3.0	0.488	0.0	0.0	2.0	0.028*	0.0	0.0	1.0	0.367
			TT	18	9.2	0.0	0.0	2.0	0.869	0.5	0.0	2.0	0.361	0.0	0.0	2.0	0.094	0.0	0.0	1.0	0.636
		Dominant	CC	96	49	0.0	0.0	2.0	Ref.	2.0	0.0	3.0	Ref.	1.0	0.0	3.0	Ref.	0.0	0.0	1.0	Ref.
			CT + TT	100	51	0.0	0.0	2.0	0.852	1.0	0.0	3.0	0.37	0.0	0.0	2.0	0.014	0.0	0.0	1.0	0.349
		Recessive	CC + CT	178	90.8	0.0	0.0	2.0	Ref.	2.0	0.0	3.0	Ref.	0.0	0.0	2.0	Ref.	0.0	0.0	1.0	Ref.
			TT	18	9.2	0.0	0.0	2.0	0.899	0.5	0.0	2.0	0.454	0.0	0.0	2.0	0.232	0.0	0.0	1.0	0.809
	rs10370	Co-Dominant	TT	113	57.7	0.0	0.0	2.0	Ref.	2.0	0.0	3.0	Ref.	1.0	0.0	3.0	Ref.	0.0	0.0	2.0	Ref.
			TG	73	37.2	0.0	0.0	2.0	0.352	0.5	0.0	3.5	0.354	0.0	0.0	2.0	0.014*	0.0	0.0	0.0	0.047*
			GG	10	5.1	0.0	0.0	2.0	0.91	2.0	0.0	2.0	0.904	0.0	0.0	2.0	0.2	0.0	0.0	1.0	0.733
		Dominant	TT	113	57.7	0.0	0.0	2.0	Ref.	2.0	0.0	3.0	Ref.	1.0	0.0	3.0	Ref.	0.0	0.0	2.0	Ref.
			TG + GG	83	42.3	0.0	0.0	2.0	0.377	1.0	0.0	3.0	0.38	0.0	0.0	2.0	0.009*	0.0	0.0	0.0	0.055
		Recessive	TT + TG	186	94.9	0.0	0.0	2.0	Ref.	1.0	0.0	3.0	Ref.	0.0	0.0	2.0	Ref.	0.0	0.0	1.0	Ref.
			GG	10	5.1	0.0	0.0	2.0	0.951	2.0	0.0	2.0	0.961	0.0	0.0	2.0	0.391	0.0	0.0	1.0	0.997

Results are from Mann-Whitney tests. * p < 0.05. Ref: reference. OHIP1 = functional limitation, OHIP2 = physical pain, OHIP3 = psychological discomfort, OHIP4 = physical disability, OHIP5 = psychological disability, OHIP6 = social disability, OHIP7 = handicap.

In the multivariate GLM analysis adjusted for sex, significant differences were found for *SOD3* gene rs2855262 polymorphism in the psychological disability domain [codominant (p = 0.045) and recessive (p = 0.038) models]. In turn, *SOD2* gene polymorphism (rs5746136) revealed a significant difference in total score [dominant model (p = 0.038)], psychological discomfort [dominant model (p = 0.034)], and physical disability [codominant model (p = 0.037)]. Finally, when analyzing the rs10370 polymorphism of this same gene, statistical differences were obtained in total score [codominant (p = 0.026) and dominant (p = 0.023) models] and handicap [codominant (p = 0.027) and dominant (p = 0.032) models]. Data from the multivariate analysis are presented in Table 4.

**Table 4:** Multivariate analysis of genotype impact on OHRQoL in General Linear Models, adjusted for sex.

Gene	SNP	Models	Genotype	General OHIP				OHIP1				OHIP2				OHIP3			

				β	SE	t	p-value	β	SE	t	p-value	β	SE	t	p-value	β	SE	t	p-value
*SOD3*	rs2855262	Co-Dominant	CC	Ref.				Ref.				Ref.				Ref.			
			CT	-2.16	1.33	-1.62	0.105	9.53	11.88	0.80	0.423	-0.42	0.29	-1.44	0.149	-0.15	0.39	-0.39	0.692
			TT	-0.32	1.68	-0.19	0.846	-0.56	14.98	-0.03	0.969	-0.12	0.37	-0.33	0.737	-0.23	0.50	-0.47	0.636
		Dominant	CC	Ref.				Ref.				Ref.				Ref.			
			CT + TT	-1.65	1.26	-1.30	0.192	6.73	11.24	0.59	0.550	-0.34	0.28	-1.22	0.220	-0.17	0.37	-0.47	0.633
		Recessive	CC + CT	Ref.				Ref.				Ref.				Ref.			
			TT	1.04	1.46	0.71	0.477	-6.58	12.96	-0.50	0.612	0.14	0.32	0.44	0.655	-0.13	0.43	-0.31	0.749
	rs13306703	Co-Dominant	CC	Ref.				Ref.				Ref.				Ref.			
			CT	-2.44	1.35	-1.80	0.072	-8.45	12.08	-0.70	0.485	-0.40	0.29	-1.39	0.165	-0.37	0.40	-0.92	0.358
			TT	-0.24	4.71	-0.05	0.958	-4.29	42.06	-0.10	0.918	0.00	1.02	0.00	0.999	-0.28	1.40	-0.20	0.841
		Dominant	CC	Ref.				Ref.				Ref.				Ref.			
			CT + TT	-2.30	1.31	-1.17	0.081	-8.19	11.75	-0.69	0.486	-0.38	0.28	-1.34	0.180	-0.36	0.39	-0.93	0.352
		Recessive	CC + CT	Ref.				Ref.				Ref.				Ref.			
			TT	0.25	4.73	0.05	0.956	-2.54	41.93	-0.06	0.951	0.08	1.02	0.08	0.934	-0.20	1.39	-0.14	0.884
*SOD2*	rs5746136	Co-Dominant	CC	Ref.				Ref.				Ref.				Ref.			
			CT	-2.06	1.20	-1.71	0.087	-10.95	10.76	-1.01	0.309	0.21	0.26	0.81	0.416	-0.68	0.35	-1.90	0.057
			TT	-3.84	2.06	-1.85	0.065	-9.14	18.49	-0.49	0.621	-0.46	0.46	-1.01	0.311	-0.89	0.61	-1.46	0.144
		Dominant	CC	Ref.				Ref.				Ref.				Ref.			
			CT + TT	-2.38	1.14	-2.08	0.038*	-10.63	10.20	-1.04	0.298	0.09	0.25	0.37	0.707	-0.71	0.33	-2.12	0.034*
		Recessive	CC + CT	Ref.				Ref.				Ref.				Ref.			
			TT	-2.89	2.00	-1.44	0.150	-4.10	17.82	-0.23	0.817	-0.57	0.44	-1.28	0.202	-0.58	0.59	-0.98	0.325
	rs10370	Co-Dominant	TT	Ref.				Ref.				Ref.				Ref.			
			TG	-2.69	1.20	-2.23	0.026*	-9.70	10.75	-0.90	0.368	0.07	0.27	0.29	0.768	-0.58	0.35	-1.63	0.104
			GG	-2.24	2.64	-0.84	0.397	-8.24	23.63	-0.34	0.727	-0.44	0.59	-0.75	0.453	-0.13	0.78	-0.16	0.865
		Dominant	TT	Ref.				Ref.				Ref.				Ref.			
			TG + GG	-2.63	1.15	-2.28	0.023*	-9.52	10.32	-0.92	0.357	0.01	0.25	0.06	0.950	-0.52	0.34	-1.54	0.124
		Recessive	TT + TG	Ref.				Ref.				Ref.				Ref.			
			GG	-1.19	2.62	-0.45	0.650	-4.46	23.24	-0.19	0.847	-0.47	0.58	-0.81	0.414	0.09	0.77	0.12	0.903
*SOD3*	rs2855262	Co-Dominant	CC	Ref.				Ref.				Ref.				Ref.			
			CT	20.36	16.72	1.21	0.224	8.64	20.21	0.42	0.669	-18.09	11.82	-1.52	0.127	-0.35	0.22	-1.56	0.120
			TT	0.84	21.09	0.04	0.968	51.37	25.50	2.01	0.045*	-17.45	14.92	-1.17	0.243	-0.23	0.28	-0.83	0.404
		Dominant	CC	Ref.				Ref.				Ref.				Ref.			
			CT + TT	14.94	15.84	0.94	0.346	20.49	19.27	1.06	0.288	-17.91	11.17	-1.60	0.110	-0.32	0.21	-1.50	0.134
		Recessive	CC + CT	Ref.				Ref.				Ref.				Ref.			
			TT	-12.01	18.28	-0.65	0.512	45.92	22.03	2.08	0.038*	-6.03	12.96	-0.46	0.642	-0.01	0.24	-0.06	0.950
	rs13306703	Co-Dominant	CC	Ref.				Ref.				Ref.				Ref.			
			CT	-13.25	17.04	-0.77	0.437	31.51	20.66	1.52	0.128	-0.83	12.08	-0.68	0.494	-0.39	0.22	-1.17	0.083
			TT	-15.96	59.32	-0.26	0.788	-0.60	71.90	-0.01	0.993	-4.42	42.06	-0.11	0.916	-0.49	0.79	-0.06	0.533
		Dominant	CC	Ref.				Ref.				Ref.				Ref.			
			CT + TT	-13.42	16.57	-0.81	0.418	29.51	20.10	1.46	0.143	-8.03	11.75	-0.68	0.495	-0.40	0.22	-1.81	0.070
		Recessive	CC + CT	Ref.				Ref.				Ref.				Ref.			
			TT	-13.22	59.16	-0.22	0.823	-7.11	72.02	-0.09	0.921	-2.71	41.90	-0.06	0.948	-0.41	0.79	-0.51	0.604
*SOD2*	rs5746136	Co-Dominant	CC	Ref.				Ref.				Ref.				Ref.			
			CT	12.17	15.05	0.80	0.419	-8.94	18.51	-0.48	0.629	-11.08	10.75	-1.03	0.304	-0.27	0.20	-1.36	0.174
			TT	54.24	25.87	2.09	0.037*	-17.17	31.82	-0.54	0.590	-9.91	18.49	-0.53	0.592	-0.25	0.35	-0.73	0.464
		Dominant	CC	Ref.				Ref.				Ref.				Ref.			
			CT + TT	19.66	14.36	1.36	0.172	-10.41	17.55	-0.59	0.553	-10.87	10.20	-1.06	0.287	-0.27	0.19	-1.41	0.157
		Recessive	CC + CT	Ref.				Ref.				Ref.				Ref.			
			TT	48.65	24.91	1.95	0.052	-13.06	30.60	-0.42	0.669	-4.83	17.83	-0.27	0.786	-0.12	0.33	-0.38	0.703
	rs10370	Co-Dominant	TT	Ref.				Ref.				Ref.				Ref.			
			TG	4.80	15.20	0.31	0.752	-4.93	18.51	-0.26	0.790	-9.78	10.75	-0.90	0.364	-0.45	0.20	-2.22	0.027*
			GG	-9.46	33.40	-0.28	0.777	-16.54	40.66	-0.40	0.684	-9.27	23.63	0.39	0.695	-0.20	0.44	-0.45	0.650
		Dominant	TT	Ref.				Ref.				Ref.				Ref.			
			TG + GG	3.07	14.60	0.21	0.833	-6.33	17.77	-0.35	0.721	-9.72	10.32	-0.94	0.347	-0.42	0.19	-2.15	0.032*
		Recessive	TT + TG	Ref.				Ref.				Ref.				Ref.			
			GG	-11.34	32.79	-0.34	0.729	-14.62	39.92	-0.36	0.714	-5.45	23.24	-0.23	0.814	-0.02	0.44	-0.06	0.951

Notes: General Linear Models were adjusted by sex. SE: standard error. *p < 0.05. OHIP1 = functional limitation, OHIP2 = physical pain, OHIP3 = psychological discomfort, OHIP4 = physical disability, OHIP5 = psychological disability, OHIP6 = social disability, OHIP7 = handicap.

## Discussion

OHRQoL is a multidimensional construct that includes a subjective assessment of an individual’s oral health, functional and emotional well-being, expectations and satisfaction with care, and sense of identity, and is an integral part of an evaluation of overall health and well-being.^21^ However, although patients’ self-perception of oral health and OHRQoL provides important complementary research and clinical data, allowing better clinical decision-making,^6,21^ few studies have analyzed this outcome in Para athletes^10^, particularly those studies involving a genetic approach. Thus, this study aimed to evaluate whether polymorphisms in genes related to oxidative stress modulate the impact on OHRQoL of Para athletes with dental caries experience. The results support our hypothesis that polymorphisms in *SOD2* and *SOD3* genes influence the OHRQoL.

Most of the Para athletes included in this study had dental caries. Studies carried out by Fernandez et al.^22^ and Pecci-Lloret et al.^23^ also observed that people with special needs, especially those with intellectual disabilities, had higher rates of dental caries and periodontal disease than population averages. Dental caries is a multifactorial, chronic, non-transmissible disease^24^ that may be associated with both lifestyle and genetic factors^25^, and that has been found to affect the OHRQoL in several populations.^26,27^ A systematic review conducted in 2017 demonstrated an association between genetic polymorphisms and the risk of dental caries for most salivary proteins evaluated.^25^ Another study by Ahmadi-Motamayel et al.^28^ stated that oxidative stress can be a biomarker for dental caries.

This study assessed dental caries in Para athletes using the DMFT index. This is a widely used epidemiological tool in oral health research to measure and compare the dental caries experience within and across populations.^19^ In the present study, the mean DMFT index as well as the overall mean of the OHIP-14 values were considered high. This may be explained by the fact that tooth decay and subsequent tooth loss can cause chewing problems, decreased appetite, sleep-related issues, and reduced performance during sports training and competition – all of which considerably affect OHRQoL.^2,10,29^ Therefore, measures to improve OHRQoL, combined with clinical and behavioral indicators, can contribute to developing and evaluating health promotion policies, joint actions, and disease prevention programs involving Para athletes.

The OHIP-14 questionnaire was used for the OHRQoL analysis. This validated tool was developed to assess the impact of oral health-related quality of life on individuals at least 14 years old, identifying their perceptions of certain dysfunctions, discomforts, and disabilities attributed to their oral condition.^18^ OHIP-14 has also been used in genetic studies^9,10^, adding to the general assessment of an individual since it has been previously pointed out that biological pathways play an important role in the general domains of quality of life.^7^

In high-performance and Para athletes, OHIP-14 scores for general quality of life and the physical domain are normally lower than for other domains. This is likely related to the pain, discomfort, and fatigue caused by intense training and the possible effects on sleep quality, dependence on medication, and physiotherapeutic treatments caused by sports injuries during their careers.^30^ However, differences in the responses of athletes and Para-athletes have been reported. Samsoiene et al.^31^ found that athletes without disabilities perceived a better global quality of life than Para athletes. In contrast, Yazicioglu et al.^32^ observed that athletes with disabilities who participated in adapted sports had a better perceived quality of life.

Superoxide dismutases are considered first-line antioxidant enzymes, which react by neutralizing the toxic effects of oxygen byproducts. In other words, they are important protective enzymes against reactive oxygen species (ROS).^33^ Usually, ROS are balanced by antioxidant mechanisms to maintain normal physiological processes. ROS constitute a critical host defense mechanism against invading pathogens.^34^ Oxidative stress is “a disturbance in the pro-oxidant-antioxidant balance in favor of the former, leading to a disruption of redox signaling and/or molecular damage”.^35^ Thus, oxidative stress may be a key factor in the development of oral diseases, especially considering that it leads to tissue destruction.^36^ Antioxidants are present in all bodily fluids, including saliva, thus preventing oxidation and protecting cells from harmful oxidants and damage caused by ROS.^37^ Therefore, infections or diseases that alter the levels of salivary antioxidants^38^, causing oxidative stress, can consequently lead to tissue destruction which can ultimately affect an individual’s quality of life.^10,38^ Lopez-Jornet et al.^16^ evaluated the relationship of salivary oxidative stress conditions and the antioxidant defense system in regards to quality of life parameters in patients with oral lichen planus, pointing towards a possible role of oxidative stress in the etiopathogenesis of this disease. However, they did not find a significant association between scores on the extended Oral Health Impact Profile-49 and the oxidative stress parameters evaluated in their study.

In this study, polymorphisms in the *SOD2* and *SOD3* genes were associated with OHRQoL scores in Para athletes with dental caries experience for the full OHIP-14 and six of its seven domains: functional limitation, psychological discomfort, physical disability, psychological disability, social disability, and handicap. To our knowledge, no studies have been conducted with this approach. However, other biomarkers have been associated with HRQoL^7^, indicating that inflammatory pathways play an important role in overall quality of life. Additionally, in our study, women reported lower values for quality of life than did men. Typically, women report more intense pain levels, more frequent pain, and a longer duration of pain than men. Furthermore, women tend to be more aware of negative physical, psychological, and social impacts on their oral health^39,40^, supporting our results.

Although our study was methodologically well designed with well-defined inclusion criteria, it has some limitations. Our analyses of Paralympic athletes did not use the disability classification system of the International Paralympic Committee. This classification system is very specific to sports-related activities seeking to reduce the consequences of an individual’s disability on their sports performance. However, our findings allow a comprehensive and integrated view of Para athletes’ oral health, thus assisting in the planning of directed oral health actions and providing a basis for future studies. Additionally, during the screening process, our protocol focused on decayed, missing, and filled teeth due to dental caries in permanent teeth, but the severity of the disease was not reported. An assessment of disease severity should be included in future studies.

This study provides new evidence on the impact of genetic polymorphisms in *SOD2* and *SOD3* genes on the OHRQoL of Para athletes with dental caries experience. However, further studies on this topic with larger sample sizes are required to improve the evaluation of these relationships.

## Conclusion

Polymorphisms in *SOD2* and *SOD3* are potentially valuable biomarkers of OHRQoL in Para athletes with dental caries experience.
